# Effect of cementation protocols on the fracture load of bilayer ceramic crowns manufactured by the Rapid Layer Technology

**DOI:** 10.1590/0103-6440202405917

**Published:** 2024-09-16

**Authors:** Sarah Emille Gomes da Silva, Júlia M. Costa Lima, Elen G. Lima, Renata M. Melo, Marco A. Bottino, Rodrigo O. Assunção e Souza

**Affiliations:** 1 Federal University of Rio Grande do Norte(UFRN),Department of Dentistry,Natal,Brazil.; 2 Institute of Science and Technology(ICT),Department of Dental Materials and Prosthodontics,São Paulo State University(UNESP/FOSJC).Brasil

**Keywords:** Aging, fracture strength, yttria-stabilized tetragonal zirconia, rapid layer technique.

## Abstract

To evaluate the fracture load of bilayer ceramic crowns manufactured by Rapid Layer Technology (RLT) after different cementation protocols of the veneering ceramic to the zirconia infrastructures. Sixty epoxy resin preparations simulating a molar tooth were obtained and 60 zirconia infrastructures and feldspathic crowns were manufactured by RLT and divided into 6 groups according to the cementation protocol at the interface to veneering ceramic (n=10): Ctr- control: conventional resin cement; Al- Al_2_O_3_ sandblasting 50µm + conventional resin cement; Al/MDP- Al_2_O_3_ sandblasting (50µm) + resin cement with MDP; Sil- silicatization 30µm + conventional resin cement; Gl/HF- glaze + hydrofluoridric acid (5%,60s) + silane + conventional resin cement; Gl- glazing as bonding agent. The feldspathic ceramic internal surface was etched with fluoridric acid (5%) + silane followed by cementation according to respective protocols. All samples were mechanically cycled (200N and 4.5x10^5^ Pa, 37°C, 2x10^6^ cycles, 3.4Hz) and submitted to axial compressing fracture load test (10kN, 0.5mm/min). The results(N) were submitted to descriptive and analytical statistical analysis with 1-way ANOVA, Weibull, and the Tukey test (α=0.05). ANOVA revealed that there was a significant difference among the tested groups (p<0.0001). The group Al/MDP presented the higher resistance to fracture (1972.46^A^ N), followed by the Ctr group (1584.41^B^ N). The characteristic strength(σ0) was significantly different (p=0.000). The crack and chipping were the predominant failures. The air-abrasion at the external surface of zirconia with Al_2_O_3_ followed by cementation with MDP resin cement, should be selected to Rapid Layer Technique when felspathic ceramic is used as veneer ceramic.

## Resumo

Este estudo tem como objetivo avaliar a resistência à fratura de coroas cerâmicas bilayers fabricadas pela técnica Rapid Layer Technology (RLT) e submetidas à diferentes protocolos de cimentação da cerâmica de cobertura sobre a infraestrutura de zircônia. Sessenta preparos de resina epóxi simulando um dente molar humano foram obtidos e 60 infraestruturas em zircônia e 60 coroas de cerâmica feldspática com 1mm de espessura na face oclusal foram fabricadas pela RLT usando um sistema CAD/CAM. As infraestruturas de zircônia foram divididas em 6 grupos de acordo com o protocolo de cimentação na interface com a cerâmica de cobertura (n=10): Ctr - controle: cimento resinoso convencional; Al - jateamento com Al_2_O_3_ 50 µm + cimento resinoso convencional; Al/MDP - jateamento com Al_2_O_3_ (50 µm) + cimento resinoso com MDP; Sil - silicatização 30 µm + cimento resinoso convencional; Gl/HF - glaze + ácido hidrofluoridrico (5%, 60s) + silano + cimento resinoso convencional; Gl - glaze como agente de união. O tratamento de superfície da superfície interna das cerâmicas feldspáticas foram condicionadas com ácido fluorídrico 5% seguidos da aplicação de silano. As cerâmicas de cobertura foram cimentadas nas infraestruturas de zircônia de acordo com seu respectivo protocolo de cimentação. Após, todas as amostras foram submetidas à ciclagem mecânica (200N e 4.5x10^5^ Pa, 37°C, 2x10^6^ cycles, 3Hz) e ao teste de resistência à compressão axial até a (10kN, 0.5 mm/min). A análise de Weibull e analyses complementares foram também realizadas. Os resultados da carga do valor máximo de resistência à fratura (N) foram submetidos à análise descritiva e estatística com ANOVA 1- fator e teste de Tukey (α = 0.05)**.** ANOVA revelou que houve diferença estatisticamente significativa entre os grupos testados (p<0.0001). O grupo Al/MDP apresentou o maior valor de resistência à fratura (1972.46^A^ N), comparado com os demais grupos seguidos pelo grupo Ctr (1584.41^B^ N) que foi similar aos demais. A resistência característica (σ0) foi significativa entre os grupos (p = 0.000). A fratura e o chipping foram as falhas predominates. O jateamento da superfície externa da zircônia com Al_2_O_3_ seguido da cimentação com o cimento contendo MDP, deve ser o protocolo escolhido para cimentação de cerâmicas feldspáticas de cobertura confeccionadas pela Rapid Layer Technique.

## Introduction

Due to its excellent proprieties, as the good mechanical strength ^1^ and biocompatibility ^2^, zirconia-based dental restorations have grown in popularity and have become the most researched material in dentistry and it's being used as a strong framework veneered with an aesthetic material ^3^. However, the veneered zirconia restorations, pressed or layered, showed a large number of veneering failures, mainly adhesive (delamination) and cohesive (chipping) fractures, that occur more often than for metal-ceramic restorations ^2^. These failures have been related to different factors as the presence or development of tensile residual stresses formed due to the thermal contraction mismatch between the zirconia and veneering ceramics ^4^.

Therefore, the bilayer crown resistance to fracture can be influenced by the veneering ceramic application technique ^2^. Currently, there are three application techniques: layered, pressed, and CADCAM. In the layered and pressed techniques, the direct application of veneering ceramic over the zirconia substructure, two material types of two different thermal coefficients of expansion (TCE) are placed into the oven for successive heating, generating great residual tension ^5^. The introduction of computer-aided design/computer-aided manufacturing (CAD/CAM) technology came as an alternative solution to this problem where the zirconia infrastructure and veneering ceramic are milled with CAD/CAM and eliminating the need for a firing step for sintering the veneering ceramic ^2^ ^,^ ^6^ ^,^ ^7^.

Using CAD/CAM technology a new alternative was introduced named the Rapid Layer technique (RLT). In this case, there are no successive ceramic sintering cycles over the zirconia infrastructure and to obtain an all-ceramic crown through this technique, the ceramic coating is cemented over the zirconia infrastructure ^5^ ^,^ ^7^. There are other techniques with the same principle as CAD/CAM Rapid Layer; however, they take low fusion glass-ceramic to bond both crown parts. In these techniques, the pre-sintered veneering ceramic is positioned over the zirconia infrastructure and is crystallized with the low-fusion glass ceramic, which is interposed between both crown parts. Studies that used this type of bonding material found higher values of resistance to fracture for crowns made with CAD/CAM technique than for the ones made with the stratified and pressed techniques ^5^ ^,^ ^6^ ^,^ ^7^ ^,^ ^8^.

In RLT it is important to have a satisfactory adhesion between the veneer ceramic and the zirconia infrastructure, and it can be achieved with the ceramic surface treatment. Dental ceramics are classified as acid-sensitive and acid-resistant, due to their surface sensibility to degradation through fluoridric acid conditioning ^9^. Although zirconia is chemically and biologically inert, so, based on the classification, suffers little or no acid degradation, because it is a silica-free ceramic and consists of a high-temperature crystal phase ^10^. Several types of surface conditioning have been tested to improve the bonding resistance between zirconia and resin cement ^9^, such as aluminum-oxide sandblasting ^11^, silicatization ^12^, surface conditioning with fluoride acid ^13^, laser radiation ^14^, silane application ^12^, primers application ^15^ and glazing ^9^.

The most common technique used to roughen the material surface and improve the bond to resin cement or porcelain is the acid treatment ^10^. As described, materials that have no glassy matrix, such as zirconia, can be treated as a glass-ceramic with the application of a layer of silica glaze to create a more reactive and etchable glass surface, which makes the ceramic able to be treated using acid conditioning and silanization, increasing bonding to resin cement ^9^. Moreover, aluminum-oxide sandblasting is also used to increase the micro-mechanical retention, through mechanical interlocking, between Y-TZP and resin cement ^11^. In the silicatization technique, particles are coated with silica and microblasted over the surface followed by silane application ^12^. Despite its excellent bonding resistance results, some studies showed that this technique can cause stress-induced transformation of zirconia ceramics, affecting the ceramic mechanical properties due to silica particles' impact against zirconia, leading to chipping propagation ^11^.

However, little information is available in the literature with in vitro studies evaluating the influence in fracture resistance of different cementation techniques between the veneer ceramic and the zirconia infrastructure manufactured by the Rapid Layer technique. This study aimed to evaluate the fracture load of bilayer ceramic crowns manufactured by Rapid Layer Technology (RLT) after different cementation protocols of the veneering ceramic to the zirconia infrastructures. The tested hypothesis was that the cementation protocol with aluminum-oxide sandblasting and the use of resin cement with MDP increased the fracture load of the crowns.

## Materials and Methods

The materials used in this study are given inBox 1.

### Preparation of the specimens (G10)

An anatomic-prepared tooth model was designed in a 3D modeling program (Rhinoceros 4.0, Seattle, WA, USA) and manufactured with epoxy resin G10 (NEMA grade G-10, International Paper, Hampton, VA, USA), corresponding to a human molar tooth prepared for a full crown (6 mm height; 1.2 mm wide chamfer, 8 mm diameter, 5.5 mm radius of curvature and 6^o^ occlusal convergence). The G10 model was duplicated with laboratory silicon (Stern Tek; Sterngold Restorative Systems) and filled with liquid epoxy resin (Huntsman A. Mat.; GmbH & Co.Kg) obtaining 60 prepared tooth models.

**Box 1:**
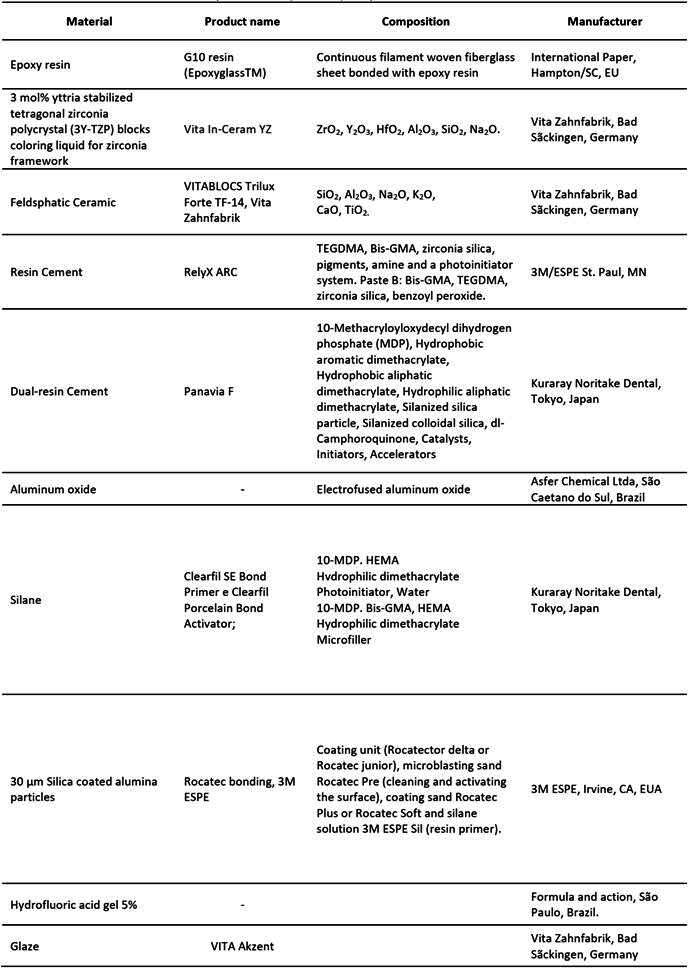
List of materials used in the study. Trade name, material, composition and manufacturer.

### Fabrication of zirconia infrastructures

The prepared resin tooth was scanned (inEos Blue; Sirona Dental Systems,) and the scan data was converted to a software (InLab 4.0 software, Sirona Dental Systems) to design a 3D image of the infrastructure zirconia coping with the Venner ceramic. After, pre-sintered partially stabilized yttrium tetragonal zirconia blocks (Vita In-Ceram YZ; Vita Zahnfabrik) were milled (CEREC MC XL InLab; Sirona Dental Systems) to obtain 60 infrastructures, which were sintered following the recommendations of the manufacturer (VITA T ZYrcomat, Vita Zahnfabrik). The occlusal surface had 1 mm of thickness after sintering processing.

### Fabrication of the veneering ceramic

The veneer ceramic crown was milled (CEREC MC XL InLab, Sirona Dental Systems) from sintered blocks of feldspathic ceramic (TriLuxe Forte TF-12, Vita Zahnfabrik) resulting in 60 feldspathic ceramic crowns with 1 mm of thickness in the occlusal surface.

The ceramic crowns (infrastructure + veneer ceramic) were subdivided according to the cementation protocol in 6 experimental groups (n=10): Ctr - control: conventional resin cement; Al/MPD - Al_2_O_3_ sandblasting (50 µm) + resin cement with MDP; Sil - silicatization 30 µm + conventional resin cement; AL - Al_2_O_3_ sandblasting 50 µm + conventional resin cement; Gl/HF - glaze, fluoride acid at 5% for 60 s and silane + conventional resin cement; Gl - glazing as bonding agent.

### Cementation protocols

Before cementation, the crowns underwent ultrasonic cleaning in isopropyl alcohol for 5 minutes, followed by drying through evaporation.

Then, the ceramic surfaces were treated according to the cementation protocols groups:

### Feldspathic ceramic surface treatment:

The feldspathic ceramic internal surface treatment followed the same procedure for Al/MPD, Sil, AL and Gl/HF groups: First the application of fluoridric acid gel at 5% (Formula and action, São Paulo, Brazil) for 60 s, washed with water/air spray, air-dried for 20 s and finished with the application of silane (Clearfil SE Bond Primer e Clearfil Porcelain Bond Activator, Kuraray Noritake Dental, Tokyo, Japan) over the conditioned surfaces.

### Zirconia infrastructure surface treatment:

### - Control (Ctr):

For Control group cementation, the resin cement (RelyX ARC, 3M ESPE) was applied inside the felspathic ceramics, which was carefully positioned over the zirconia infrastructure. Then, a load of 750 g was applied perpendicular to the crown for 1 minute to ensure a uniform flow and a thin cement layer. Afterward, excess cement was removed, and each face of the light of the crown was polymerized for 40 s (1200 mW/cm², Radii-Cal, SDI Limited, Victoria, Australia).

### -Al/MPD:

Using a specific blasting device and a 50 μm Al_2_O_3_ (Asfer Chemical Ltda, São Caetano do Sul, Brazil) particle, zirconia surfaces were abraded at a pressure of 2.5 bar at a distance of 15 mm for 10 s. The cementation was performed with PANAVIA F 2.0 (Kuraray Medical Inc.) which contains MDP, so primers are not mandatory. The resin cement was applied on the inner side of felspathic ceramic and positioned over zirconia infrastructure, also under a load of 750 g as previously described. Afterward, excess cement was removed, and each face of the crowns was light cured for 40 s (1200 mW/cm2, Radii-Cal, SDI Limited, Victoria, Australia).

### -Sil:

The air abrasion was performed at the zirconia surface with 30 µm silica-coated aluminum-oxide particles**,** for 10 seconds following the standardized method as described for the Al/MDP group (15 mm distance and 2.5 bar pressure). Then, a silane layer (Clearfil SE Bond Primer and Clearfil Porcelain Bond Activator; Kuraray Medical Inc.) was applied over the surfaces.

### -AL:

The AL Group had the surface treated only with 50 µm aluminum-oxide sandblasting, as described for the Al/MDP group, before the cementation. After, the crowns underwent ultrasonic cleaning with isopropyl alcohol for 5 minutes and drying through evaporation.

### -Gl/HF:

Gl/HF Group had the cementation protocol with the application of a thin layer of low-fusion glass-infiltrated ceramic and sintered**,** followed by the application of fluoridric acid gel at 5% for 60 s in the area where low-fusion ceramic was applied. After fluoridric acid removal, the silane was applied.

The cementation protocol for Sil, AL, and Gl/HF groups was performed with the conventional resin cement, RelyX ARC (3M ESPE). The cement was applied on the inner side of feldspathic ceramic which was positioned over zirconia infrastructure under the same device with a load of 750 g as previously described**.** Excess cement was removed, and each face was photopolymerized for 40 s (1200 mW/cm², Radii-Cal, SDI Limited, Victoria, Australia).

### -Gl:

For the Gl group, glaze (VITA Akzent, Vita Zahnfabrik, Bad Sãckingen, Germany) was applied (powder and liquid mix) on the inner side of the veneer ceramic and then positioned over zirconia infrastructure with uniform and constant pressure. This protocol has the objective of simulating the technique suggested by the CAD-ON system, pioneered by Ivoclar®, where two components, the zirconia framework and the glass-ceramic veneer were manufactured utilizing CAD-CAM technology and these components are subsequently fused with a glass, yielding a trilayer ceramic restoration ^16^. After the excess glaze was removed, the crowns were taken to an oven and submitted to the glaze firing cycle.

### Crown cementation over epoxy resin preparations

The epoxy resin models were etched with 5% HF on the cementation surfaces for 60 s ^17^, washed with water jets for 20 s, and air-dried. Next, a silane layer (Clearfil SE Bond Primer and Clearfil Porcelain Bond Activator; Kuraray Medical Inc.) was applied over the conditioned surfaces and, after 60 seconds, they were treated with a primer mixture (ED Primer, Kuraray Medical Inc).

The resin cement (Panavia F 2.0, Kuraray Medical Inc.) was applied on the inner side of the zirconia infrastructure and positioned over the epoxy resin preparation under a load of 750 g. Excess cement was removed and each face was photopolymerized for 40 s (1200 mW/cm², Radii-Cal, SDI Limited, Victoria, Australia).

### Mechanical cycling

All crowns were mechanically cycled (Erios 11000; ERIOS Equipamentos Técnicos e Científicos Ltda) with a load of 200 N applied to the center of the main groove of the occlusal face of the crown through round-tipped stainless steel piston with 6 mm diameter, for 2.10^6^cycles, at 3.4 Hz. During cycling, samples were immersed in water at 37ºC, with temperature control via an integrated thermostat. Every 200,000 cycles, crowns were analyzed in the stereo microscope for chipping presence evaluation, at 70x magnification by a single calibrated operator.

### Fracture load test

Each sample was submitted to axial compressing fracture load testing (DL 1000; EMIC) with a load tip (stainless steel, 6 mm diameter) located in the center of the main groove of the occlusal face of the crown. The test was carried out with a load cell of 10kN (0.5 mm/min) until fracture.

### Statistical Analysis

The sample’s power was calculated through the website www.openepi.com by comparing the higher and the lower means and standard deviation of fracture load data considering a 95% confidence interval and the sample size of 10 specimens in each group. Data distribution was obtained on fracture resistance caused by the axial compression test and was assessed by Shapiro-Wilk's test and homogeneity by Levene's test. The results indicated normal distribution and equality of variances (p > 0.05). The maximum fracture load strength results (N) were subjected to the Dunnett test (5%), ANOVA 1-way, and Tukey test (α=0.05). For these analyses, the computer program Statistix (Analytical Software Inc., version 8.0, 2003) was used.

The Weibull modulus and characteristic strength (mean and 95%CI) were determined from equation 1 where F is the failure probability, σ0 is the initial strength, σc is the characteristic strength, and m is the Weibull modulus; Characteristic strength is the strength in which the probability of failure is approximately 63%.

$$\ln\left(1/1-f\right)=m\ln\sigma_{c}-m\;ln\;\sigma_{0}$$

Equation 1 **.** Weibull modulus calculation

### Fracture mode analysis

For the failure mode analysis, all crowns subjected to compression strength test were analyzed with a stereo microscope (70x, Discovery V2; Zeiss), and fractures were classified as crack-cracking of the veneering ceramic at the interface; chipping - fracture on the surface of the veneering ceramic without exposure of the framework; delamination - fracture of the veneering ceramic with exposure of the framework; and catastrophic - fracture of the veneering ceramic and zirconia framework ^18^.

## Results

### Resistance to compression

ANOVA 1-way revealed that there was a significant difference among tested groups (p<0.0001) and the Tukey test showed that the Al/MDP group (1971.1 ± 274.0^A^ N) had the higher fracture load, being statistically different from the other groups (Table 1).

No crown from the Gl group was submitted to mechanical cycling and to fracture load test, as this technique did not produce viable crowns because neither zirconia nor the veneering ceramic bonded to the glaze.

The Weibull modulus (m) has no statistical difference (P= 0.875) and characteristic strength (σ0) was significantly different among groups (p = 0.000). The Al/MDP (2089.14^a^ N) group showed the highest characteristic strength (σ0), which was statistically similar to Ctr (1725.64^ab^ N) and different from the Gl/HF (1667.42^b^ N), AL (1533.4^b^ N) and Sil (1284.54^b^ N). Weibull analysis results are described inTable 1 andFigure 1.

**Figure 1:**
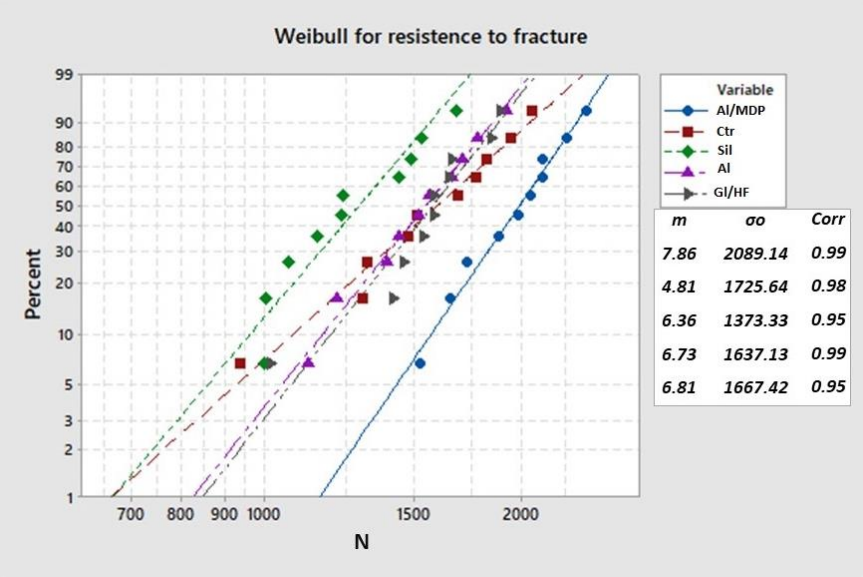
Weibull plot of resistance to fracture (n=10).

**Table 1:** Mean Fracture Load (N) with standard deviation, characteristic strength (σ_o_), Weibull modulus (*m*) and respective CI (95%) for Resistance to fracture caused by axial compression of experimental groups (n=10).

Group name	Surface treatment	Fracture Load (N)	Weibull Characteristic strength (σo) (N)	95% CI for (σo) (N)	Weibull Modulus (*m*)	**95% CI for *m***
Ctr (control)	No	1584.41 ± 340.29^B^	1725.64^ab^	1506.33 - 1976.88	4.81	2.4 - 9.4
AL/MDP	Aluminum-Oxide Sandblasting + Resin Cement With MDP	1972.46 ± 274.33^A^	2089.14^a^	1922.71 -2269.98	7.86	4.4 - 14.0
Gl/HF	Glaze + Fluoridric Acid + Silane	1563.52 ± 243.43^B^	1667.42^b^	1514.45 - 1835.85	6.81	3.3 - 13.6
AL	Aluminum-Oxide Sandblasting + Relyx ARC	1533.4 ± 247.22^B^	1637.13^b^	1485.80 - 1803.86	6.73	3.8 - 11.9
Sil	Silicatization + Relyx ARC	1284.54 ± 238.14^B^	1373.33^b^	1238.31 - 1523.07	6.36	4 - 10

*The Tukey test (p < 0.05). Different upper case letters show statistical differences between groups in the same column. Different lower case letters show statistical differences between groups on Weibull Characteristic strength.

### Fracture mode analysis

**Figure 2:**
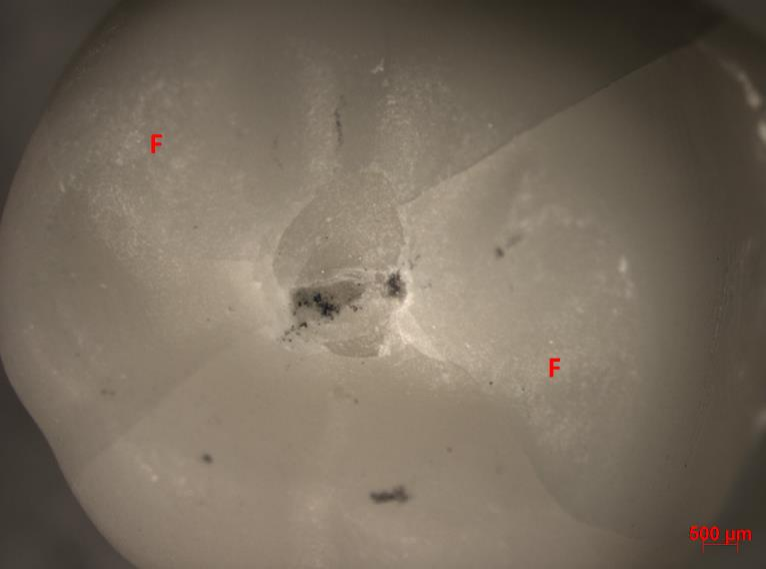
Representative image of crack fracture (AL Group, 10X). F = feldspathic veneering ceramic

**Figure 3:**
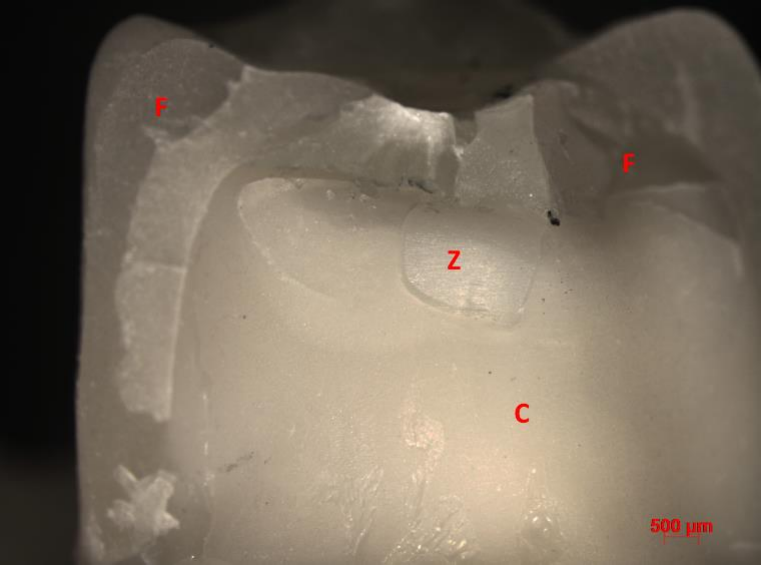
Representative image of delamination fracture (Ctr Group, 10X). F = feldspathic veneering ceramic; Z = zirconia framework; C = resin cement.

In terms of fracture mode, overall, the crowns were intact after mechanical cycling. On the other hand, after the fracture load test, the predominant failure among groups was cracking (62%), followed by delamination (26%). The Al/MDP group exhibited the highest percentage of catastrophic failures among the groups (30%), followed by the Al group (20%). Chipping failures were not found for tested groups (Table 2, Figures.2,3, and4).

**Table 2:** Fracture mode classification

Group	Crack	Chipping	Delamination	Catastrophic fracture
Ctr (control)	6 (60%)	0 (0%)	3 (30%)	1 (10%)
Al/MDP	5 (50%)	0 (0%)	2 (20%)	3 (30%)
Gl/HF	5 (50%0	0 (0%)	5 (50%)	0 (0%)
AL	8 (80%)	0 (0%)	0 (0%)	2 (20%)
Sil	7 (70%)	0 (0%)	3 (30%)	0 (0%)
TOTAL	31 (62%)	0 (0%)	13 (26%)	6 (12%)

**Figure 4:**
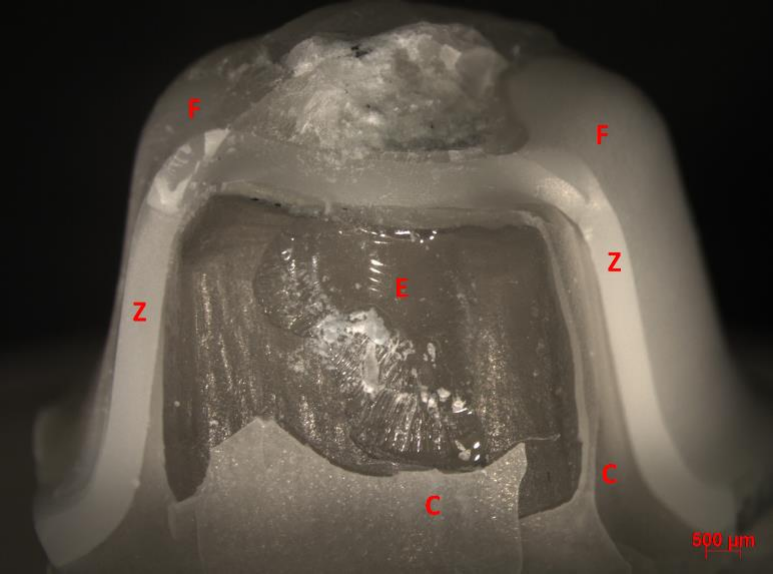
Representative image of catastrophic fracture (AL Group, 10x). F = feldspathic veneering ceramic; Z = zirconia framework; C = resin cement; E = epoxy resin.

## Discussion

In the current in vitro study, the fracture load with different cementation techniques of veneering ceramic to zirconia infrastructure with CAD/CAM Rapid Layer technique was investigated. On RLT the machined veneer is adhesively bonded to zirconia infrastructure using a resin composite luting agent. Studies have shown that this technique optimizes fatigue behavior and increases fracture load compared with hand-layered ceramics, mainly because the technique eliminates the firing steps, which prevents the development of residual stress ^6^ ^,^ ^8^. Previous studies have reported promising results regarding the mechanical properties and stability of crowns using veneering ceramic manufactured by the RLT technique. Tom et al. (2023) found a higher fracture load for crowns fabricated with RLT, with a mean ranging from 2680 N to 2560 N depending on the thickness analyzed, while the pressed group exhibited a fracture load of 1400 N ^8^. Consistent with these findings, the present study found a fracture load of up to 1972.46 N for crowns manufactured with RLT and cemented with MDP-based resin cement. On the other hand, conventional techniques such as layered core-veneer and heat pressing have been well evaluated in the literature for their mean fracture loads ^4^ ^,^ ^6^ ^,^ ^7^. Lima et al. (2020) ^7^ reported fracture loads for the pressed technique ranging from 3941.5 N to 4608.9 N under different cooling protocols and for layered crowns, a mean of 2942.9 N to 3232.0 N ^7^.

Nevertheless, previously Schmitter et al. ^19^ compared the resistance of CAD/CAM Rapid Layer crowns with those pressing and stratified techniques and observed that RLT presented an initial lower fracture resistance result. These authors believed that these results could be related to interposed material (resin cement) between both ceramic structures. The Finite Element Analysis (FEA), revealed that tension similar to ceramic material resistance reached the resin cement internal surface, causing fracture with low load application. However, the study also revealed that zirconia frameworks veneered with CAD/CAM-produced feldspathic ceramic are less sensitive to aging than zirconia crowns with layered feldspathic veneer ^19^. Riedel and collaborators (2019) ^5^, showed that the study groups produced using RLT did not show any chipping event during the testing period, showing a superior fatigue behavior of machined and adhesively bonded veneers over hand-layered reinforced-glass veneers ^5^. Moreover, studies have demonstrated that employing industrial prefabricated blocks in conjunction with the milling technique, utilizing the same ceramic material as the conventional method leads to an increase in the Weibull modulus of oxide ceramics. Consequently, this enhances the reliability of restorations. It can be concluded that sintering a CAD/CAM-milled veneer cap to the zirconia core significantly enhances mechanical stability ^8^.

The findings of the study showed that the tested hypothesis was accepted, and the different cementation protocols influenced the resistance of the crowns. Achieving a stronger bonding strength without the decrease of mechanical proprieties of the materials is an important parameter in terms of the clinical success of any restoration. The surface treatment used can influence the mechanical performance of the bilayer ceramic crowns ^20^. For the Rapid Layer technique, the manufacturer`s cementation protocol indicated a cement that contains MDP, PANAVIA (Kuraray Medical Inc.), the results of the present study showed that the group treated with this cement (Al/MDP) had superior resistance to the others groups. This leads us to believe that the fragility of the tested system is in the use of resin cement for veneer ceramic/zirconia bonding. The Weibull analysis corroborated with the findings of the resistance to compression test, where the Al/MDP group showed the highest characteristic strength and was similar to the Ctr group.

Several studies investigated the more efficient cementation methods and the effect of sandblasting over zirconia structure ^10^ ^,^ ^11^ ^,^ ^21^, the best results of bonding resistance were observed in sandblasted surfaces (air abrasion) and cemented with primer and/or resin cement with MDP ^11^. The MDP cement monomer ester phosphate directly bonds to metallic oxides, as the zirconia oxide ^21^, the studies show that when the sandblasting and the resin cement with MDP techniques are used a better bonding strength is observed ^10^. When comparing the bonding resistance of conventional and MDP-resin cement, the cement with MDP showed more bonding resistance ^22^, which can directly influence the mechanical resistance of the crown. The literature indicates that the type of cement used impacts the distribution of stresses, aiding in to dissipate occlusal forces away from the tooth-restoration interface. This is crucial as ceramic restoration fractures can originate at the intaglio surface or cementation interface, where tensile stresses are concentrated ^23^. This highlights the importance of achieving a strong bond between the ceramic restoration and the resin cement to reduce susceptibility to failure ^21^. In this study, the zirconia surface treatment analysis was prioritized. For this reason, conventional cement was used in all groups, except for one, in which the manufacturer’s indicated protocol was applied. Hence, it was possible to observe that, more important than the adopted surface treatment, is the type of applied resin cement, as this was the only group with statistically significant differences if compared to the other groups.

The densely-sintered sandblasted ceramic improved micromechanical retention due to the increase in surface roughness, increasing superficial energy and wettability ^11^. Araújo et al. (2018) ^12^ observed that, when comparing different zirconia surface treatments, the treatment that presented the best results on bonding resistance to zirconia was the silicatization followed by the use of a silane coupling agent. The sandblasting with Al_2_O_3_ coated by silica is associated with a higher transformation from a tetragonal to a monoclinic phase, generating compressive stress that opposes cracking propagation ^11^. In the present study, the group treated with silicatization presented the lower fracture load results and, for not presenting statistically different values from groups with conventional non-MDP cement, the use of conventional cement may have been a determining factor for the obtained low compression resistance results.

To improve the performance of these restorations the mechanical properties of the interface material are taken into consideration. An important property of resin cement that clinically influences cemented restoration longevity is the elasticity modulus. Cement with adequate elasticity modulus may bear elevated occlusal loads and when the elasticity modulus is elevated, a better performance and lasting of all-ceramic crowns is observed ^24^. The elasticity modulus of low fusion glass-ceramic is around 70 GPa, according to its manufacturer; the elasticity modulus of the Panavia F 2.0 resin cement is 18.3 GPa, and of the Relyx ARC cement is 9.6 GPa ^24^. Fixed dental prostheses are more likely to be subjected to bending forces than to other types of stresses, because of this the flexural properties of resin cement are also important and are closely associated with the material's composition. Duymus et al. (2013) found that the flexural strength of Bis-GMA-based composite resin cement is higher than that of the other cement, where PANAVIA F presented higher flexural strength than RelyX ARC cement, which is in good agreement with the results of the present study ^25^.

It is important to understand the failure mechanics of dental ceramics to develop stronger ceramic materials. The cement interposed between the ceramic restoration and the tooth and, in this study’s case, between the zirconia infrastructure and the ceramic coating, affects the propagation of cracks in the inner portion of the ceramic crown. The small defects and micro-cracks of ceramic restorations are filled by the resin cement, inhibiting crack propagation and increasing the restoration's resistance to fracture ^23^. There is no relation between the type of fracture and the cementation mode. Delamination and catastrophic fracture are the most severe types of fracture and even groups that bore higher loads presented such fracture types. Clinically, the predominant type of fracture for RL restorations is still unknown.

Based on the parameters evaluated in this study, conclusions summarize that the air-abrasion at the external surface of zirconia with Al_2_O_3_ followed by cementation with MDP resin cement, should be selected to Rapid Layer Technique when felspathic ceramic is used as veneer ceramic.
